# Systematic comparison of lncRNAs with protein coding mRNAs in population expression and their response to environmental change

**DOI:** 10.1186/s12870-017-0984-8

**Published:** 2017-02-13

**Authors:** Qin Xu, Zhihong Song, Caiyun Zhu, Chengcheng Tao, Lifang Kang, Wei Liu, Fei He, Juan Yan, Tao Sang

**Affiliations:** 1 Key Laboratory of Plant Resources and Beijing Botanical Garden, Institute of Botany, Chinese Academy of Sciences, Beijing, 100093 China; 2 State Key Laboratory of Systematic and Evolutionary Botany, Institute of Botany, Chinese Academy of Sciences, Beijing, 100093 China; 3 University of Chinese Academy of Sciences, Beijing, 100049 China; 4 Biology Department, Brookhaven National Laboratory, Upton, NY 11973 USA; 5 Key Laboratory of Plant Germplasm Enhancement and Speciality Agriculture, Wuhan Botanical Garden, Chinese Academy of Sciences, Wuhan, Hubei 430074 China

**Keywords:** Population transcriptome, lncRNAs, *Miscanthus lutarioriparius*, Co-expression, Environmental response, Expression diversity

## Abstract

**Background:**

Long non-coding RNA (lncRNA) is a class of non-coding RNA with important regulatory roles in biological process of organisms. The systematic comparison of lncRNAs with protein coding mRNAs in population expression and their response to environmental change are still poorly understood. Here we identified 17,610 lncRNAs and calculated their expression levels based on RNA-seq of 80 individuals of *Miscanthus lutarioriparius* from two environments, the nearly native habitats and transplanted field, respectively.

**Results:**

LncRNAs had significantly higher expression diversity and lower expression frequency in population than protein coding mRNAs in both environments, which suggested that lncRNAs may experience more relaxed selection or divergent evolution in population compared with protein coding RNAs. In addition, the increase of expression diversity for lncRNAs was always significantly higher and the magnitude of fold change of expression in new stress environment was significantly larger than protein-coding mRNAs. These results suggested that lncRNAs may be more sensitive to environmental change than protein-coding mRNAs. Analysis of environment-robust and environment-specific lncRNA-mRNA co-expression network between two environments revealed the characterization of lncRNAs in response to environmental change. Furthermore, candidate lncRNAs contributing to water use efficiency (WUE) identified based on the WUE-lncRNA-mRNA co-expression network suggested the roles of lncRNAs in response to environmental change.

**Conclusion:**

Our study provided a comprehensive understanding of expression characterization of lncRNAs in population for *M. lutarioriparius* under field condition, which would be useful to explore the roles of lncRNAs and could accelerate the process of adaptation in new environment for many plants.

**Electronic supplementary material:**

The online version of this article (doi:10.1186/s12870-017-0984-8) contains supplementary material, which is available to authorized users.

## Background

The genome complexity of higher eukaryotes is revealed by a high proportion of non-coding for proteins regulatory elements, of which a class of processed long non-coding RNA (lncRNA) is attracting increasing attention. The lncRNAs are highly heterogeneous transcripts in length, varying from 200 bp to tens of thousands of nucleotides. They pervasively distribute in genomes, not only in intron and intergenic region, but also in some antisense transcripts, pseudogenes and retrotransposons [1]. LncRNAs have lower level of sequence conservation than protein-coding mRNAs [2]. They were originally considered as transcriptional by-products or “expression noise” of protein coding genes and were often dismissed in analyses of transcriptome [3], however recent studies uncovered the increasing body of evidences that lncRNAs strictly regulated and played important roles in biological process of organisms [4].

Recently, a great number of lncRNAs had been identified in animals and humans [5–11]. They participated in numerous biological processes, such as X-chromosome inactivation [12], and human diseases [6–8, 13, 14], although only a few of them have been functionally annotated. Compared with researches about lncRNAs in animals and humans, studies on plants are relatively infrequent, and only restrict to some model plants, such as *Arabidopsis*, rice, maize, and *Populus* [15–19]. Most of the annotated lncRNAs are related with regulation of the development of plants.

LncRNAs are found to be differentially expressed between different organs, development stage and environment. The fact that lncRNAs are expressed in a strong state-specific manner was revealed by the recent analyses of lncRNAs expression under abiotic stress conditions [20–22]. Thus it is reasonable to identify the lncRNAs associated with stressful environment by comparing the lncRNA expression pattern across environments. Indeed, the identified differentially expressed lncRNAs during abiotic stress had suggested the important role of lncRNAs in plant stress response [17]. To date, some powdery mildew infection and heat stress-responsive lncRNAs were identified in wheat [23], and some lncRNAs induced by drought, high salt, cold, and abscisic acid were identified in *Arabidopsis* [24]. These studies provide us a good starting point of understanding the role of lncRNAs in the process of abiotic stress tolerance in plants [21].

Although several studies have identified plant lncRNAs that are activated under various environmental conditions, only a few of them have been characterized in population under field condition. Due to the low level of expression frequency and strong state-specific expression manner of lncRNAs, only a limited number of lncRNAs can be identified using RNA-seq for a few samples, and the components that are important in specific condition may been overlooked. Genome and population wide approaches could provide a larger scale of lncRNAs identification and a broader picture of the role of lncRNAs as regulators of the massive protein coding genes responding to stress under field environment.

With markedly accelerated consumption of fossil energy and its worsening environmental impact, the development of energy crops to provide renewable feedstock for clean energy and safe material offers an appealing solution to the sustainability problems facing the society [25]. The extent to which this can solve the problem depends heavily on whether the crops can be produced in large scales without threat to the food security and at little cost of natural ecosystem function [26]. A promising candidate of dedicated energy crops meeting these requirements is *Miscanthus* [25, 27], a group of C4 perennial grasses capable of producing high biomass on marginal land [28]. The challenge now becomes whether and how the new crop can adapt to meet environmental requirements. The integration of population genetics and new genomic technologies holds a great potential to meet the challenge.

Of more than a dozen wild *Miscanthus* species, *M. lutarioriparius* that produces the highest biomass stood out as a desirable wild progenitor for crop adaptation. Fourteen populations of *M. lutarioriparius* were collected across the distributional range of this endemic species in central China and were planted in two experimental fields in 2009, one located aside its native habitat in Jiangxia of the Hubei Province (JH) and the other located in Qingyang of the Gansu Province (QG) in the Loess Plateau [29]. Loess Plateau, one of the most seriously eroded regions of the world, possesses remarkably large area of marginal land for producing second-generation energy crops such as *Miscanthus* [28]. It has been shown that *M. lutarioriparius* was not only able to establish in QG but also to produce higher biomass than in JH, while the annual precipitation and temperature is nearly two-third and 10-degree lower in QG than in JH [29–36].

In this study, we used population RNA-seq data to identify lncRNAs in *Miscanthus* leaves and to investigate their roles in adaptation to environmental change. Here, we obtained a comprehensive list of 17,610 lncRNAs. Systematic expression analysis revealed that the expression of lncRNAs displayed more variation than protein coding mRNAs. Integrating with our published datasets of water use efficiency (WUE) [32], we highlighted potential contributions of lncRNAs to improve WUE and adaptation to environmental change.

## Results

### *De novo* assembly and identification of putative lncRNAs

We performed RNA-seq to investigate 80 individuals of *Miscanthus* transcriptomes. Non-directional paired-end RNA-seq was carried out and ~2.76 billion 80 bp paired-end reads were generated after quality control. Population transcripts were assembled using the Pipeline PopART which combined multiple *kmers* and multiple individuals [33, 37]. Totally we obtained 818,491 transcripts, out of which 18,503 unique mRNAs from the 80 individuals were identified by blasting against the latest annotation of transcriptome references of close related species including *Sorghum bicolor*, *Zea mays*, *Oryza sativa*, and *Brachypodium distachyon*. To identify lncRNAs, we used Coding Potential Calculator software to evaluate the protein-coding potential for transcripts longer than 200 bp [38], and transcripts with the evidence of protein-coding potential were discarded. All the remaining transcripts were aligned against Rfam database to discriminate lncRNAs from previously annotated miRNA, rRNA, or other small noncoding RNA transcripts [39]. To obtain a more reliable data of lncRNAs and a better comparison between two environments, we discarded the lncRNA candidates that expressed in less than 20 individuals out of the 80 individuals. Under this criterion, a total of 17,610 putative lncRNAs were remained. This amount was similar to that of lncRNAs in *Arabidopsis*, rice and maize [16, 24].

**Fig. 1:**
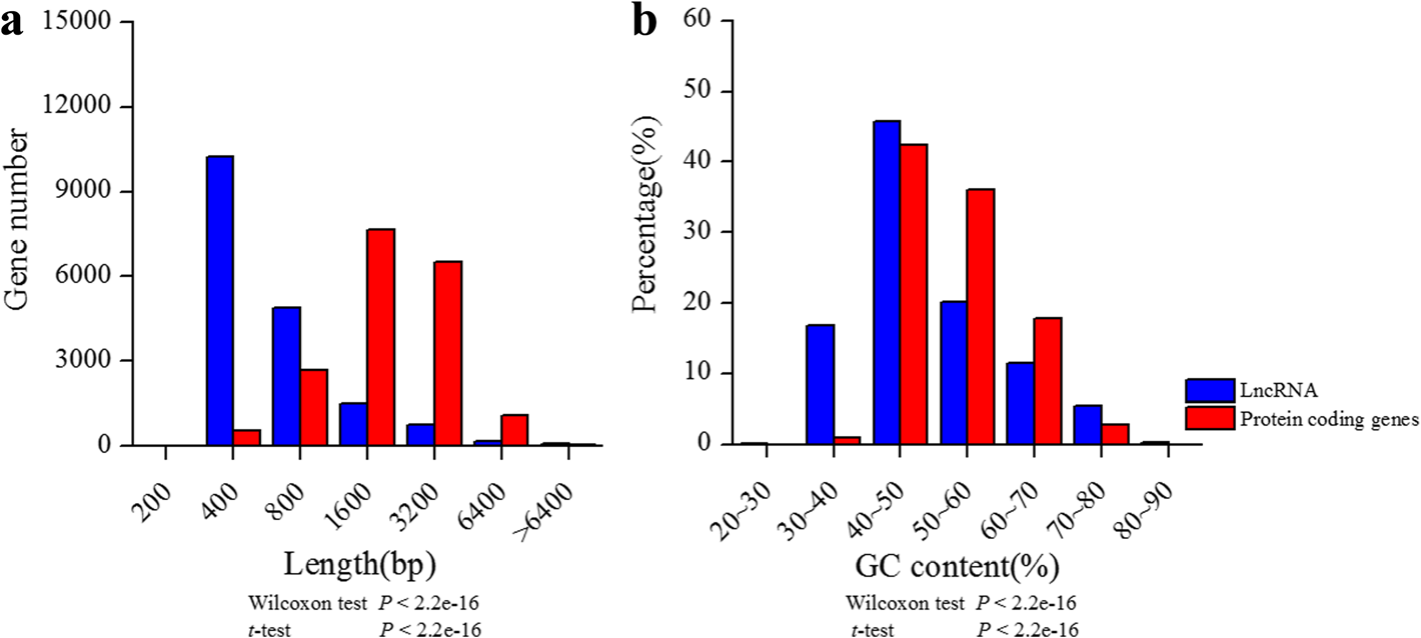
Sequence characterization of lncRNAs compared with mRNAs. **a** The distribution of sequence length. **b** The distribution of GC content

The lengths of lncRNAs ranged from 200 to 5196 bp, the majority (92%) of which were around 200–600 bp (Fig. 1a and Additional file 1). The length of lncRNA was 683 bp in average and 352 bp in median, respectively, which was longer than those in *Arabidopsis* [24]. The length of protein coding mRNAs was 1601bp in average and 1425bp in average respectively. LncRNAs were significantly shorter than mRNAs in length (Wilcoxon test or *t*-test, *P* < 2.2e-16; Fig. 1a). In addition, GC content for lncRNAs and mRNAs were 46% and 51% in median, respectively. The lncRNAs showed significant lower GC content than mRNAs (Wilcoxon test or *t*-test, *P* < 2.2e-16; Fig. 1a and Additional file 1). In order to examine whether these candidate lncRNAs sequence assembly were reliable, 8 lncRNAs were randomly selected and validated by full-length PCR experiments. PCR results showed that the sequence identity ranged from 94% to 100% compared with the reference sequences assembled from population data (Table 1 and Additional file 2). As the reference sequences of these lncRNAs were assembled from the population transcriptome data, there could be some single nucleotide polymorphisms or indels (insertions and deletions). For example, the sequence of lncRNA_MluLR14524 by PCR method has an 84bp deletion compared with the reference sequence derived from with RNA-seq, which may be due to an alternative splicing of this individual or a chimera between two similar lncRNAs in the assembling data. In general, the results suggested a high quality assembly of the lncRNAs and they could be used for further analysis.

**Table 1:** Summary of the PCR validation for assembled lncRNAs

LncRNA	Assemble length	PCR length	Coverage	Identity
lncRNA_MluLR14385	960	907	94%	98%
lncRNA_MluLR14524	748	625	93%	94%
lncRNA_MluLR6328	687	593	86%	99%
lncRNA_MluLR15001	538	393	73%	99%
lncRNA_MluLR14468	586	516	88%	99%
lncRNA_MluLR14141	699	360	51%	100%
lncRNA_MluLR2250	781	281	35%	100%
lncRNA_MluLR6864	499	69	13%	100%

### Higher expression diversity of lncRNAs compared with that of mRNAs within and across environments

**Fig. 2:**
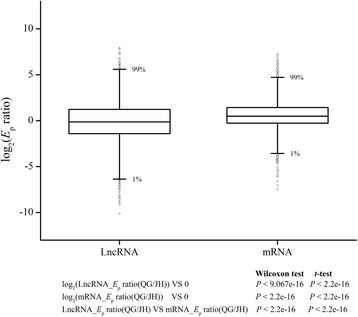
Boxplot of log_2_(*E*_p_ ratios) of lncRNAs and mRNAs. *E*_p_ ratio was calculated with *E*_p_(QG)/*E*_p_(JH). The Box represents 25%–75% frequency interval. The whiskers extend to the range of 1% and 99% in plot. Outliers were also plotted as individual points. The following figures were in accordance with the same criteria

We first set out to characterize the global expression pattern of lncRNAs in JH and QG. The expression level of lncRNAs in population (*E*_p_) was calculated. Then, the expression levels of lncRNAs and those of protein coding mRNAs were compared. It was found that *E*_pS_ of lncRNAs in QG shifted significantly toward to lower levels, suggesting an overall depression of lncRNAs expression from JH to QG. The change of lncRNAs expression between two environments was examined by *E*_p_ ratios of QG to JH for each lncRNA, and it was found that the resulting distribution of log_2_(*E*_p_ ratios) deviated significantly from 0 and skewed toward bottom (Wilcoxon test or *t*-test, *P <* 2.2e-16; Fig. 2 and Additional file 3), indicating that lncRNAs decreased expression level in QG. Compared with protein coding mRNAs, lncRNAs had significantly lower *E*_p_ ratios (Wilcoxon test or *t*-test, *P* < 2.2e-16; Fig. 2, Additional files 3 and 4). Meanwhile, lncRNAs had a wider range of *E*_p_ ratios than that of protein coding mRNAs (Fig. 2, Additional files 3 and 4).

**Fig. 3:**
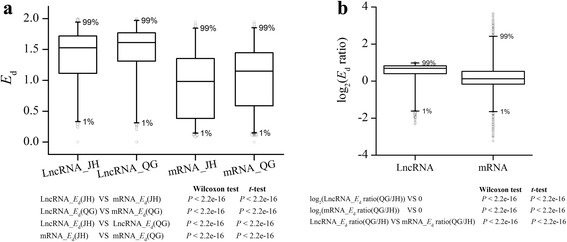
Expression diversity of lncRNAs compared with mRNAs. **a** Boxplot of expression diversity of lncRNAs and mRNAs in JH and QG. **b** Boxplot of log_2_(*E*_d_ ratios) of lncRNAs and mRNAs. *E*_d_ ratio was calculated with *E*_d_(QG)/*E*_d_(JH)

To evaluate and compare population variation of lncRNAs expression within and between environments, we calculated and compared expression diversity (*E*_d_) between JH and QG. Compared with protein coding mRNAs, lncRNAs had significantly higher *E*_d_ in both environments (Wilcoxon test or *t*-test, *P* < 2.2e-16; Fig. 3a, Additional files 3 and 4). Moreover, the distributions of *E*_d_ of lncRNAs were compared between two environments, and the result showed that *E*_d_ were significantly higher in QG than in JH with 60.3% of *E*_d_s being elevated in QG (Wilcoxon test or *t*-test, *P* < 2.2e-16; Fig. 3, Additional files 3 and 4). The significant differentiation was also detected by *E*_d_ ratios between JH and QG for each lncRNA, and log_2_(*E*_d_ ratios) of lncRNAs were compared with 0, and the distribution of log_2_(*E*_d_ ratios) significantly deviated from 0 and skewed toward top (Wilcoxon test or *t*-test, *P* < 2.2e-16; Fig. 3b, Additional files 3 and 4). In addition, the *E*_d_ ratios of lncRNAs were significantly higher than protein coding mRNAs (Wilcoxon test or *t*-test, *P* < 2.2e-16; Fig. 3b, Additional files 3 and 4). The higher expression diversity of lncRNAs may suggest a relaxed expression regulation compared with protein coding mRNAs.

**Fig. 4:**
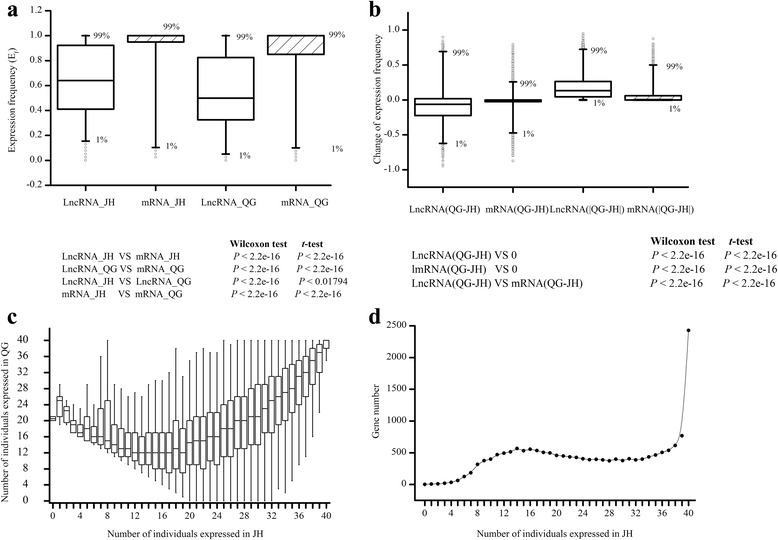
Expression frequency of lncRNAs in population compared with mRNAs. **a** Boxplot of expression frequency of lncRNAs and mRNAs in population in JH and QG. **b** Boxplot of the change of expression frequency of lncRNAs and mRNAs from JH to QG. **c** Boxplot of the number of individuals that expressed lncRNAs in JH and QG. Boxplot was used for visualization purpose. **d** The number of lncRNAs that expressed in JH with different individuals

To evaluate the expression variation of lncRNAs in the population, we measured the expression frequency for each lncRNA in two environments. It was found that lncRNAs expressed at significantly lower frequency than protein coding mRNAs in both environments (Wilcoxon test or *t*-test, *P* < 2.2e-16; Fig. 4a and Additional file 5). Expression frequency in QG showed a lower level than JH for both lncRNAs and mRNAs (Wilcoxon test, *P* < 2.2e-16; *t*-test *P <* 0.01794; Fig. 4a and Additional file 5), suggesting an overall decrease of both lncRNAs and mRNA expression frequency from JH to QG. The change of lncRNAs and mRNAs expression frequency between two environments was examined by difference of QG to JH for each lncRNA and mRNA, respectively. It was found that lncRNAs had a broader range of the difference than mRNAs (Wilcoxon test or *t*-test, *P* < 2.2e-16; Fig. 4b and Additional file 5). The lower level and larger difference of expression frequency for lncRNAs might suggest the loosen expression regulation compared with protein coding mRNAs.

### Differential expression of lncRNAs in environment responsive regulation

Emerging evidences showed that lncRNAs participate in stress responsive regulation when plants are in the face of stressful environment [15, 23], thus we analyzed the differential expression and variation of lncRNAs between the two environments. First, we plotted the number of individuals in which lncRNA expressed between two environments (Fig. 4c). Only 1376 (7.8%) of the 17610 lncRNAs expressed in all individuals (Fig. 4d and Additional file 5), and only 45 out of the 80 individuals could be detected the lncRNA expression in median (Additional file 5), suggesting the specific and differential expression manner of lncRNAs in our population. Specially, it was found that 2 and 59 lncRNAs were detected only in QG and JH (Additional file 5), respectively. These newly arisen lncRNAs in those regions or environment-specific lncRNAs may be related with environmental adaptation. It was also possible that they just lost from the other region and had no function. In addition, except the low frequency of lncRNAs which expressed in less than 10 individuals in JH, the expression frequency of lncRNAs between 2 environments was positively correlated (Fig. 4c), suggesting that most of these lncRNAs expressed at robust or similar expression frequency between environment, and also suggesting the accuracy of the estimation of lncRNAs expression.

On the other hand, the differentially expressed genes between two sites were tested to identify candidate genes responding to environmental stress. The expression level changes (*E*_p_ ratios) of 17,610 lncRNAs between two environments were calculated. The magnitude of fold change (log_2_) in the expression level of lncRNAs under new environment was observed between −9 to 10.8 (Additional file 3). We obtained a total of 2,063 lncRNAs that were over 2- fold up- or down- regulated with FDRs less than 0.01, including 821 up-regulated and 1242 down-regulated lncRNAs (Additional file 3). Because genes with substantially increased expression diversity in new stress environment QG could have increased phenotypic variation upon which natural and artificial selection could act to improve adaptability of the *Miscanthus* species, we also calculated the expression diversity changes (*E*_d_ ratios) of lncRNAs between two environments for lncRNAs. The magnitude of fold change in the expression diversity of lncRNAs under stress condition ranged from 0.12 to 6.65 (Additional file 3). A total of 931 stress responsive lncRNAs with *E*_d_ ratio of more than 2-fold change were obtained. These lncRNAs may have higher potential in regulation of genes expression for contribution to adaptation.

**Fig. 5:**
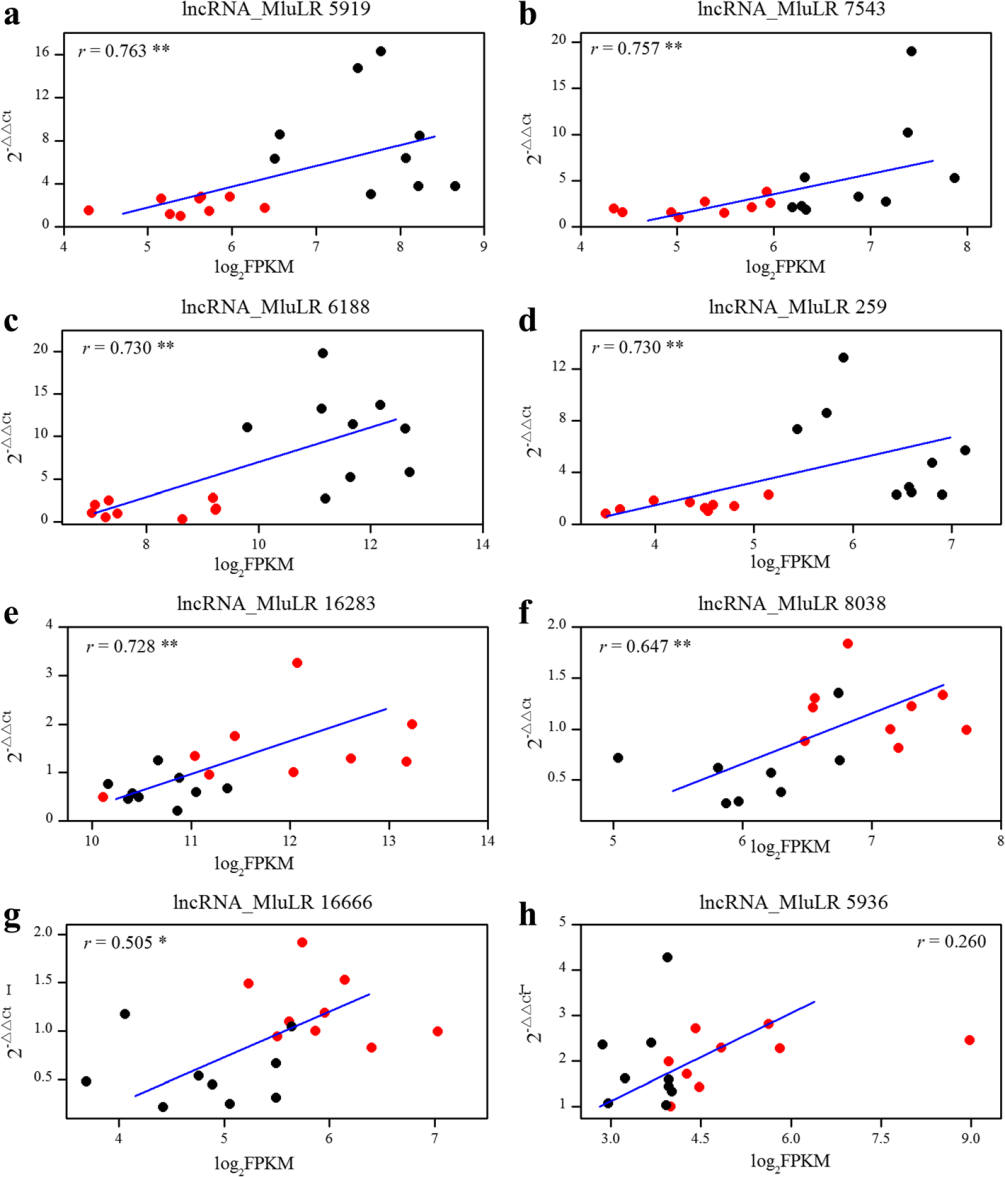
Expression level correlation between RNA-seq and qPCR for 8 lncRNAs (**a**-**h**). Each of the lncRNA name was shown on the top of figure. The x-axis denotes the FPKM value quantified by RNA-seq, while the y-axis shows the relative expression value obtained by qPCR. Positive correlation between FPKM values of RNA-Seq and the relative expression value of qPCR indicate a consistent estimation of the relative expression levels between the two methods. The r value in the graphs indicates the correlation coefficient. ** represents the significant level (*P* < 0.01, Spearman’s rank correlation test). Sequences of qPCR primers are given in Additional file 10. Black and Red dots represent individuals sampled from near native site JH and the transplanted site QG, respectively

To validate the reliability of the expression level, quantitative real-time PCR (qPCR) was performed to assay the accuracy of the RNA-seq by using 9 individuals from each field site for 8 lncRNAs which were differentially expressed between two environments. The relative quantitation of expression levels which was calculated using 2 ^–ΔΔCt^ method was compared with the FPKM values in RNA-seq data in each sample [32, 40–42] (Fig. 5). It was found that the relative expression levels determined by the two methods were significantly correlated except for lncRNA_MluLR5936 which had outlier value in 2 samples (Fig. 5). The results confirmed that the relative gene expression levels were reliable and could be used for further analysis.

### Functions of differentially expressed lncRNAs based on lncRNA-mRNA co-expression network

In order to explore the correlation of lncRNAs and mRNAs, we used the transcripts with *E*_d_ more than 0.6 to perform pairwise correlation. In total, 3,086 lncRNA-mRNA pairs in both environments had the correlation coefficient of more than 0.9 (*P <* 0.001; Table 2 and Additional file 6), which included 1,431 mRNAs and 1,601 lncRNAs, respectively. These lncRNA-mRNA expression pairs which would represent the consensus or robust relationship between environments were constructed, and the max number of node was 8 for mRNAs and 33 for lncRNAs. These results suggested that lncRNAs in the robust relationship may play more pivotal roles in the process of regulation network. In addition, there were 215,251 lncRNA-mRNA pairs having the correlation coefficient of more than 0.95 in QG but smaller than 0.1 in JH. Similarly, there were 241,459 lncRNA-mRNA pairs having the correlation coefficient of more than 0.95 in JH but smaller than 0.1 in QG. These differentially co-expressed lncRNA-mRNA pairs may involve in environment-specific regulation (*P <* 0.001; Table 2).

**Table 2:** Pairwise number of lncRNA-mRNA co-expression in the two environments

Parameter	Pairwise number	lncRNA number	mRNA number	Max nodes of lncRNA	Max nodes of mRNA	Implication
R(JH) > 0.9&R(QG) > 0.9	3086	1601	1431	33	8	Robust relationship between environment
R(JH) > 0.95&R(QG) > 0.95	929	598	553	14	7	
R(JH) > 0.95&R(QG) < 0.7	290705	6783	4180	396	241	Environmental response-related relationship
R(QG) > 0.95&R(JH) < 0.7	267035	7203	4245	317	179	
R(JH) < 0.1&R(QG) > 0.95	215251	6957	4096	308	169	
R(QG) < 0.1&R(JH) > 0.95	241459	6633	4059	374	227	

**Fig. 6:**
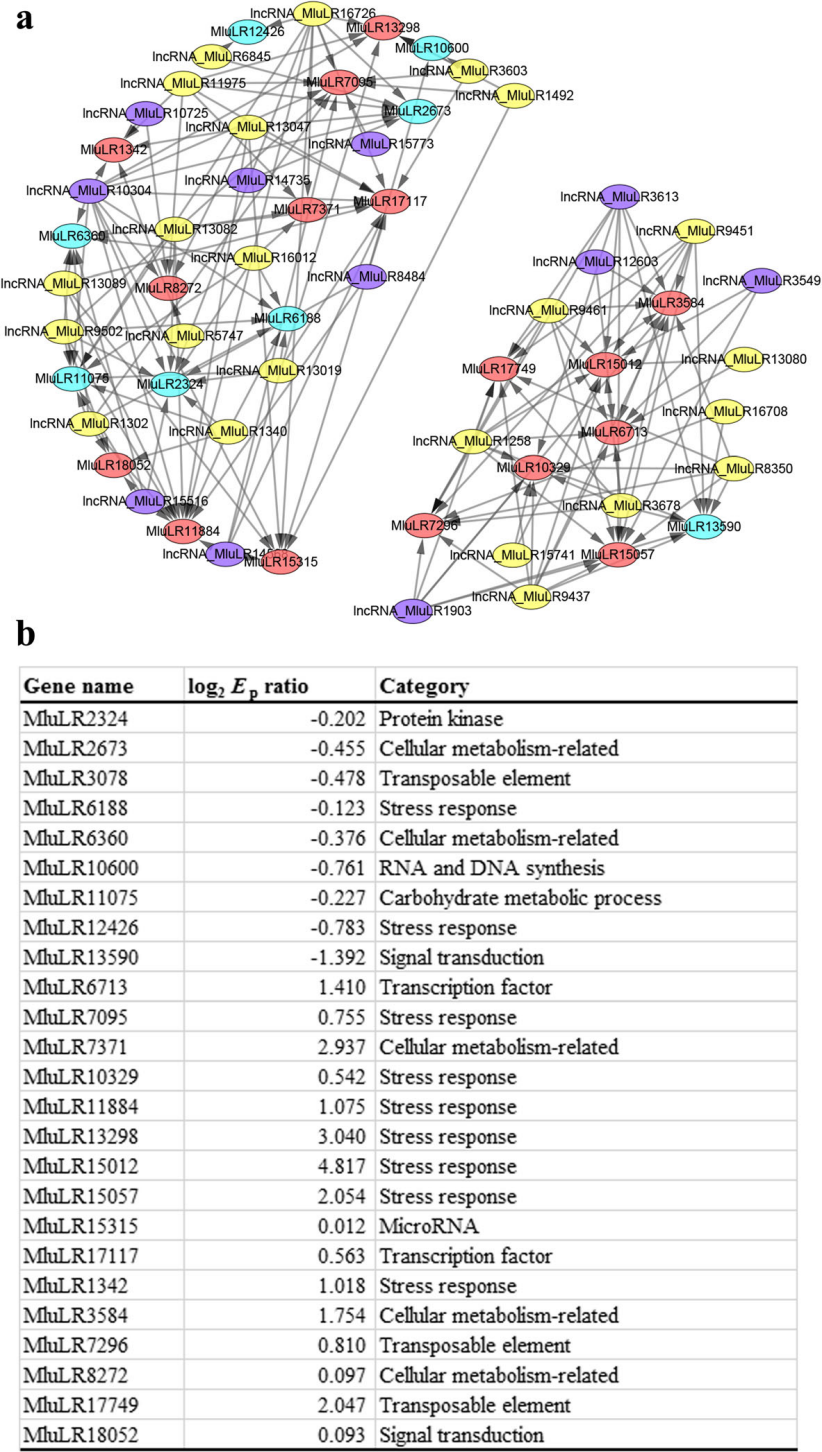
**a** Visualization of environment responsive differential lncRNA-mRNA co-expression network. The nodes with red, blue, purple or green colors represent up-regulated mRNAs, down-regulated mRNAs, up-regulated lncRNAs and down-regulated lncRNAs. The size of node positively related with connection degree. **b** The fold change value and the functional categories of 25 mRNAs in the co-expression network

The environmental-specific lncRNA-mRNA co-expression network between two environments can be used to identify key lncRNAs responding to environmental change. As genes with increased expression diversity in new environment may be relevant with adaptation, we filtered out 2003 lncRNAs and 4108 mRNAs with *E*_d_ ratio larger than 1.5. Finally 2 272 lncRNA-mRNA pairs with Spearman correlation coefficients larger than 0.7, including 1 052 mRNAs and 1 023 lncRNAs (Additional file 7), were identified for network construction. The similar amount of lncRNAs and mRNAs potentially responding to environmental change suggested that lncRNAs may play the roles as important as mRNAs in environmental adaptation. Two sub-networks containing 38 lncRNAs and 25 mRNAs with lncRNA-mRNA connection degree more than 10 were presented in detail (Fig. 6a). Out of the two sub networks, 12 and 26 lncRNAs were found to be up-regulated and down-regulated respectively, while 16 and 9 mRNAs were up-regulated and down-regulated respectively. Our network showed that one lncRNA could co-express with multiple mRNAs and one mRNA could be correlated with multiple lncRNAs. The network implied a complex relationship between lncRNAs and mRNAs.

To reveal potential functions of the identified lncRNAs, we analyzed Gene Ontology (GO) terms and families of protein coding mRNA genes associated with the stress-responsive lncRNAs due to their regulating roles (Additional file 7). We detected significant enrichments of 17 families with more than 5 members and 8 GO terms in leaves under stressful environment. For examples, we found 5 categories of genes that Protein kinase domain, Ring finger domain, Cytochrome P450, WD domain, G-beta repeat had more than 12 members (Table 3). Based on the functional categories from the Pfam domain annotations, nine of the 25 protein coding mRNAs in the two sub-networks were found to be related with stress response (Fig. 6b).

**Table 3:** The enrichments of families of the protein-coding genes identified by the environmental-specific lncRNA-mRNA co-expression network

Family	NO.
Protein kinase domain	13
Ring finger domain	13
Cytochrome P450	12
WD domain, G-beta repeat	12
RNA recognition motif. (a.k.a. RRM, RBD, or RNP domain)	9
alpha/beta hydrolase fold	7
EF-hand domain pair	7
Alpha/beta hydrolase family	6
DnaJ domain	6
Protein phosphatase 2C	6
AMP-binding enzyme = Domain of unknown function (DUF4009)	5
Glutathione S-transferase, C-terminal domain = Glutathione S-transferase, N-terminal domain	5
GRAS family transcription factor	5
Myb-like DNA-binding domain	5
PPR repeat = PPR repeat family	5
Sugar (and other) transporter	5
Zinc finger, C3HC4 type (RING finger)	5

### Identification of key lncRNAs regulating water use efficiency (WUE)

**Fig. 7:**
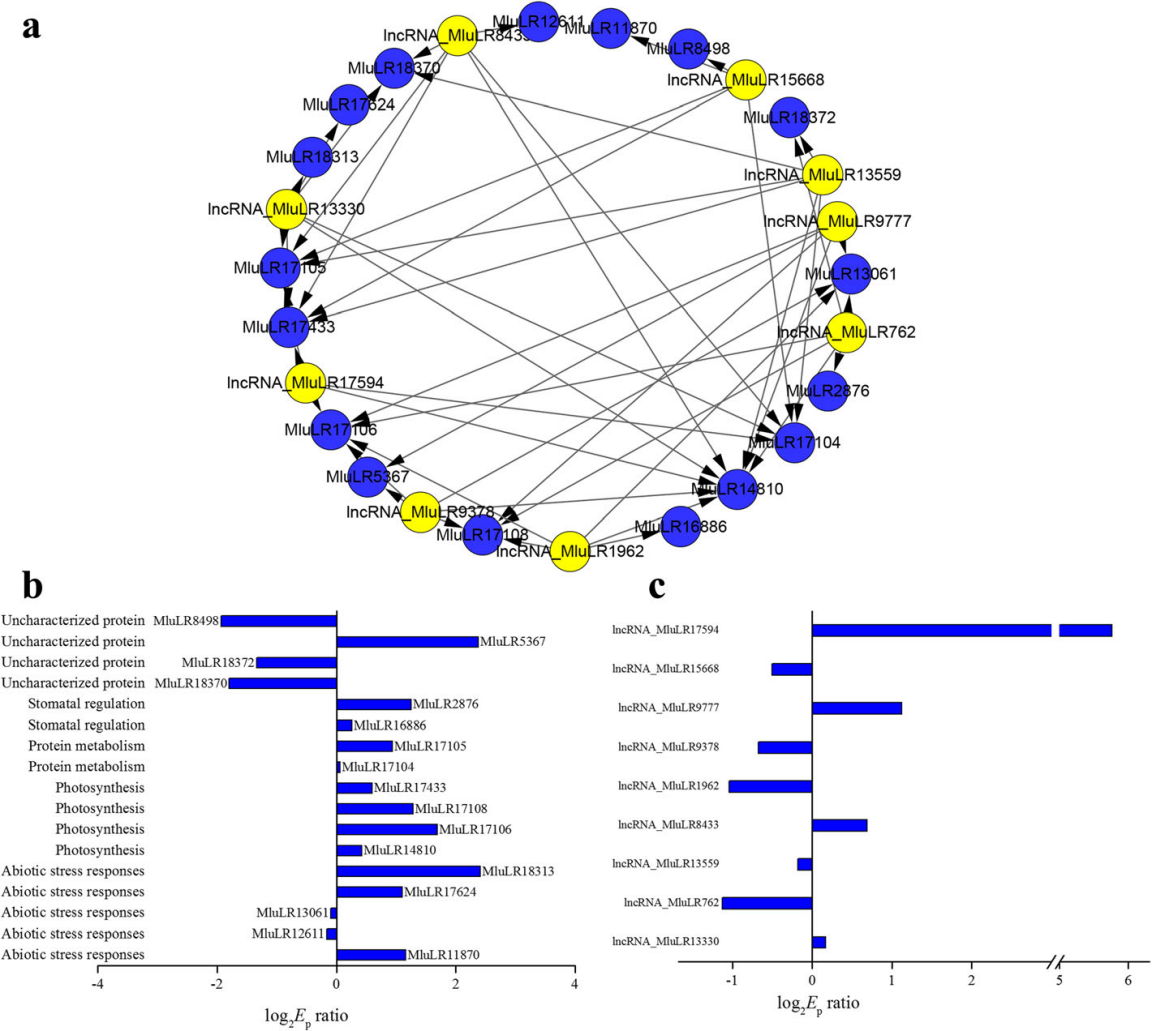
**a** Water use efficiencies (WUE)-related differential lncRNA-mRNA co-expression network with connectivity level larger than five. The yellow square nodes represent the mRNA, the blue square nodes represent lncRNA. Edges connecting two nodes have a direction associated with them. **b** The functional categories and the fold change value of 17 mRNAs. **c** The fold change value of 9 lncRNAs

Previous genome-wide association studies found hundreds of lncRNAs containing trait-associated SNPs, suggesting the putative contributions of lncRNAs to agricultural traits [16, 43], and we had identified 48 candidate genes whose expression were related with WUE in our previous study [32]. To find out and reveal the potential contribution of lncRNAs to WUE, we analyzed co-expression of lncRNAs and mRNAs using Spearman correlation coefficient for each candidate gene of WUE, and constructed the WUE-lncRNA-mRNA co-expression network (WUE-LMN). As a result, this co-expression network contained a total of 48 candidate genes, 3371 unique lncRNAs and 4277 edges (Additional file 8). The degree of the candidate genes ranged from 23 to 215, indicating that each of these genes associated with adaptation was regulated by complex network involving multiple lncRNAs. We further filtered the co-expression network with more than five nodes for lncRNAs (Fig. 6a). Nine lncRNAs and 17 candidate genes for WUE were remained. And the remained 17 candidate genes for WUE were mainly functioned in abiotic stress responses, photosynthesis and stomatal regulation (Fig. 7b). Of the 9 lncRNAs, 4 and 5 lncRNAs were up-regulated and down-regulated between two environments,respectively (Fig. 7c). We inferred that they may represent hub genes or regulators in WUE related pathway and played an important role in responding to environmental changes.

## Discussion

### The reliability of lncRNAs identification

Since *de novo* assembly is disadvantaged by its inability to account for incidents of structural alterations of mRNA transcripts, such as alternative splicing, *de novo* assembly of lncRNAs reference without a known genome faces even more huge difficulties due to lack of sequence conservation [2]. Here we reported population transcriptome-wide identification of lncRNAs with high reliability. We substantially improved the accuracy of the *M. lutarioriparius* lncRNAs assembly. First, we perform multiple assemblies with various *kmer* lengths, which could reach the best trade-off between the length and quantity of transcripts [44]. In addition, we used population transcriptome data to retain the best part of each one to form the final assembly [37]. The complementary effect of multiple individuals completed the transcriptome assembly in both transcript number and length [33]. Second, although lncRNAs often express in a low frequency, lncRNAs that expressed in less than 25% of individuals were filtered out. This procedure could greatly reduce the assembly errors and improve the reliability of the remained lncRNAs. Moreover, the sequences were further validated by PCR sequencing and the expression level of lncRNAs was proved by qPCR. The results suggested the sequence of lncRNAs was high-quality assembled and the expression level was accurately evaluated. Nevertheless, we would demonstrate that a small proportion of the identified lncRNAs may be not real in our data, as non-coding transcripts derived from transposable elements were not filtered out due to the lack of a sequenced genome from *M. lutarioriparius*. In summary, the population-wide RNA-seq assembly approach for identifying lncRNAs was proven as an efficient method and could be applied for other species.

### Relaxed expression regulation of lncRNAs and more sensitive to environment compared with protein coding mRNAs

In our study, we found the expression diversity of lncRNAs were significantly higher than that of protein coding RNAs in both JH and QG, the two independent strictly controlled experiments (Fig. 3a). This result may be explained by that population of *Miscanthus* harbored more substantial variation in noncoding regions of the genome, including extensive genetic variation within cis-regulatory elements and transcription factor binding sites. The results also implied that lncRNAs may be loosely regulated compared with protein coding mRNAs [45]. This may be due to that lncRNAs do not directly function and a large number of lncRNAs are nonfunctional, and thus changes in most lncRNA expression exact little fitness cost [5]. Higher-level expression diversity of lncRNAs may provide the indirect evidence for that lncRNAs experienced more relax evolution or divergent selection than protein coding mRNAs.

Previous results that lncRNA expression is typically more variable between tissues [5, 46, 47], which may be an indicator that the expression diversity of lncRNAs in population could be larger than mRNAs. Based on this inference, lncRNAs may be more variable among individuals, i.e. preferential expression in some individuals, but the majority of the mRNAs were consistently expressed in all individual [5]. This hypothesis could be illustrated by the comparison of expression frequency between lncRNAs and protein coding mRNAs (Fig. 4). Expression diversity provides raw materials for adaptation, because it represents a source of variation may enrich phenotypic variation at the level influencing more closely than genetic diversity and consequent fitness under natural selection [33]. Thus the lncRNAs and protein coding mRNAs with higher expression diversity had the higher potential as a variation factor in contribution to phenotypic variation [15]. Moreover, the lncRNAs had even more higher potential to be the variation factor than compared to protein coding mRNAs due to their higher variation of expression frequency (Fig. 4).

It intuitively seems that the range and average value of expression diversity ratio of lncRNAs between JH and QG are wider and higher than that of mRNAs, respectively (Fig. 3). The widened ranges of expression levels and larger expression diversity suggest the lncRNAs are more sensitive to environmental change than mRNAs. As lncRNAs can epigenetically regulate the protein coding mRNAs by interacting with cis or trans elements [1, 17, 48], the increased expression diversity of lncRNAs might have triggered the mechanisms of enriching the expression diversity of genes in response to the environmental change [33]. Moreover, studies on adaptation in wild populations had revealed that ecologically important phenotypes changed due to the transcriptional regulation [49–51]. Thus, the increased diversity of lncRNAs may contribute to the change of ecologically important phenotypes such as WUE in our study and play an important role in improving the adaptation to new environment [43]. Thus, the lncRNAs with increased expression diversity could be the potential target contributing to adaptation.

### Important roles of lncRNAs in regulation of stress response and agricultural trait

Previous studies have demonstrated that after stimulation with stress, expression level and expression diversity for some genes or transcription factors related to stress response were elevated [33, 52]. However, the expression variation for lncRNAs had been little reported after stress stimuli. Here we found that the expression level of 807 lncRNAs (4.6%) and 995 mRNAs (5.4%) altered more than two-fold (Additional files 3 and 4), which suggested the proportion of lncRNAs with high expression fold change is comparable to mRNAs. Among the lncRNAs that we identified, we found that 2086 lncRNAs (11.8%) may be related with stress condition based on the *E*_d_ ratio, which reveal that extent of expression variation between two environments [33]. In particular, 76 lncRNAs had *E*_d_ ratio of more than 5-fold changes, which exhibited strong stress-responsive expression pattern [33, 53]. Moreover, 52 lncRNAs expressed at the level of nearly 100-fold increase from JH to QG. These lncRNAs with extreme change of expression could have the potential roles in adaptation to stress [17].

A large number of genes which were highly correlated with lncRNAs were identified using mRNA-lncRNA co-expression network analysis and their GO term enrichment analysis [54, 55]. And 5 categories of genes were closely related to cellular stress response using GO term enrichment analysis (Table 2). Protein kinase domain played a role in a multitude of cellular processes, and Protein kinase pathway mediated drought and cold signaling [56]. Ring finger domain mainly involved in ubiquitination pathway and abiotic stress responses [57]. Cytochrome P450 mainly involved in biosynthetic reactions and biotic stress [58]. Functions of WD domain, G-beta repeat ranged from signal transduction and transcription regulation to cell cycle control, autophagy and apoptosis, and they may response to salt stress and nutrient stress [59–61]. These enrichment pathways could have been related with the cold and dry climates and poor soil conditions in the Loess Plateau. These results suggested that lncRNAs could have played the roles in many biological processes responding to stress through regulating gene network.

The higher WUE was likely to be one reason for the higher biomass production with much less precipitation in more stressful environment [30]. Using co-expression network analysis, we identified 3371 unique lncRNAs involving in the regulation of these candidate genes, which revealed a regulation pattern of lncRNAs in the manner of the accumulation of numerous minor lncRNAs for complex traits. Out of these lncRNAs, 9 lncRNAs regulated at least 5 candidate genes. This suggested that lncRNA has the potential to be the regulation center in the wide-range participation. Furthermore, the environmental change from JH to QG involves intricate natural condition, including soil water content, light condition, temperature etc. [30, 33]. Complex co-expression network regulation could be one of the most important molecular mechanisms underlying the adaptation to these conditional changes [9, 15, 22]. The evidence presented in this study indicates that only a small proportion of lncRNAs with large expression variation has an impact on phenotypic variation in WUE. It is reasonable to infer that much more lncRNAs may participate in the response to other conditional changes. Here we revealed the regulation pattern of lncRNAs for complex traits [43], which provided a good starting point towards understanding the role of lncRNAs in regulation of abiotic stress tolerance.

Although we identified a large number of lncRNAs related with many biological process, a limited number of lncRNAs had been speculated their functions using lncRNA-mRNA co-expression analysis. Further studies are necessary to understand the functional motifs of lncRNAs, and how specific lncRNAs seek out selective sites in the genome for lncRNA-mRNA and lncRNAs-lncRNAs interaction.

## Conclusion

In this study, we identified population-wide lncRNAs in *M. lutarioriparius* under two different field conditions. The expression level and the variation of expression of lncRNAs in population were systematically characterized and were compared with protein coding mRNAs. We proposed that lncRNAs may experience more relaxed evolution or more divergent selection compared with protein coding RNAs. In response to new environment, lncRNAs may be more sensitive to environmental change than protein coding RNAs. This study would provide insights into the roles of lncRNAs in plant stress responses. Such information can be useful in the process of adaptation of second generation of energy crop.

## Methods

### Data collection

We had collected populations of *M. lutarioriparius* across their distributional ranges along the Yangtze River in China in October and November 2008, and planted them in two experimental fields. One field site was in Jiangxia of Hubei Province (JH) which was near the native habitat and established by Wuhan Botanical Garden of Chinese Academy of Sciences, and the other field site was in Qingyang of Gansu Province (QG) which was near the domestic habitat and established by the Key Laboratory of Plant Resources and Beijing Botanical Garden, Institute of Botany, Chinese Academy of Sciences. QG is colder, drier, and nutrient-poorer condition than its native habitat. The voucher specimen was deposited in the Wuhan botanical garden of Chinese Academy of Sciences (HIB). We then collected the same 14 populations of *M. lutarioriparius* in nearly native habitat JH and target domestic site QG in 2012 [33]. The growing stage was about one month later in QG than in JH, which was consistent with the temperature patterns between the two locations [29]. Therefore, samples which were listed in Additional file 9 were collected around noon on June 12^th^, 2012 in JH and on July 13^th^, 2012 in QG, respectively. When these plants were at the same growth stage between the two sites, 3 individuals for each population were randomly chosen and the fourth mature leaves of these plants were sampled for RNA-seq using Hiseq 2000 [33]. Considering the quality of reads, 2 individuals whose Q20 and genes expression level deviated from that of the majority of the individuals in both sites were discarded. Thus a total of 80 individuals were used for transcriptomic analysis ultimately. The raw data had been released at NCBI’s Short Read Archive under three Bio Projects, PRJNA227191, PRJNA227195, and PRJNA226258. The transcriptome sequencing data, which was previously used in protein coding mRNAs expression analysis was proved to be a high quality of transcriptome reference [33].

### Transcriptome assembly

We formed two assembly groups by randomly pooling 24 and 30 individuals from the 80 sampled individuals of *M. lutarioriparius. De novo* assembling was performed on SOAPdenovo-Trans for each group respectively [62], the average insert size was set at 230 bp, while the mergeLevel set at 0 to force perfect match. The coverage for contigs less than 3 was eliminated. The threshold value of output number of scaffolds was set at 15. The program GapCloser was used for filling the gaps of scaffolds with the sequence overlap length set at 25.

For each of the assembly group, *kmer* 41, *kmer* 51, and *kmer* 61 were used during assemble and all the resulting scaffolds were pooled [44]. The pooled scaffolds were merged when possible and joined together for extending sequence using the program CAP3 [63]. The minimal overlapping length was set at 45 bp and the overlapping identities of from 94% to 99% were tested. The scaffolds obtained from SOAPdenovo-Trans and merged scaffolds obtained from CAP3 for the two assembly groups were pooled together. The ORFs of all these sequences were predicted by the EMBOSS package [64], and only sequences with ORFs smaller than 150 bp were retained.

### Identification and characterization of lncRNAs

All transcripts over 200 bp were filtered from raw assembled transcriptome for coding potential evaluation using Coding Potential Calculator software (http://cpc.cbi.pku.edu.cn/programs/run_cpc.jsp) [38], a sequence-based predictor for protein coding potential of transcripts and a widely used discoverer of lncRNAs. The negatively scored transcript was considered a noncoding transcript. To discriminate lncRNAs from previously annotated miRNA, rRNA, or other small noncoding RNA transcripts, we aligned all noncoding transcripts against the Rfam database [39]. The remaining transcripts were identified and were treated as candidate lncRNAs of *Miscanthus*, which was used for further functional analysis. The RNA sequencing reads from the 80 individuals were aligned independently against the lncRNA candidates. To gain enough sequencing data for lncRNA assembly, we combined all aligned results for each lncRNA candidate using SAMtools [65]. The mapped reads were then assembled into transcripts using Cufflinks [66]. The assembled transcripts with sequence length less than 200 bp were filtered to remove. To obtain a good comparison between two environments, we discarded the lncRNA candidates expressed in less than 20 individuals out of the 80 individuals. Under this criterion, 17610 lncRNA candidates were remained. The GC content as well as sequence length was compared between lncRNAs and protein coding mRNAs using both *t*-test and Wilcoxon test.

### Differential expression of lncRNAs and mRNAs

Expression level of lncRNA was estimated based on transcript abundance calculated using FPKM. FPKM value for both genes and lncRNAs was calculated using Cufflinks. Reads of each individual were mapped to the reference transcriptome or lncRNA using Bowtie, TopHat, and Cufflinks [67, 68]. Reference sequence index was created using bowtie-build, and the short reads were aligned to the reference genes and lncRNAs sequence using TopHat using default settings. In this study, we used *E*_d_ which is the abbreviation of “expression diversity” as a way to estimate the Standard Deviation. Expression level and population expression diversity were calculated based on the formula ${E}_{\mathrm{p}}=\frac{{\displaystyle \sum_{i=1}^n{E}_{\mathrm{i}}}}{n}$ and ${E}_{\mathrm{d}}=\frac{{\displaystyle \sum_{i=1}^n\left|{E}_{\mathrm{i}}-{E}_{\mathrm{p}}\right|}}{\left( n-1\right){E}_{\mathrm{p}}}$, here *E*_i_ represents the FPKM of a given gene or lncRNA of the *i*th individual in the population, *n* represents the number of individuals, and *E*_p_ represents the expression level of a given gene or given lncRNA [33]. *E*_p_ ratio and *E*_d_ ratio, which represents the fold change of *E*_p_ and *E*_d_ from JH to QG respectively, and *E*_d_ were compared between lncRNAs and protein coding mRNAs as well as between environments using both *t* test and Wilcoxon test. In order to more accurately detect candidate genes responding to new environmental stress, we used both significant test and a fold change ranking [69, 70]. The differentially expressed genes between the two sites were identified using the parametric *t*-test (normal bimodal distribution) or the non-parametric Wilcoxon-test (non-normal unimodal distribution). And gene expression change of lncRNA with FDRs less than 0.01 and larger than two-fold change was considered to be statistically significant.

### Construction of the lncRNA-mRNA co-expression network

LncRNA-mRNA co-expression network was constructed following a method similar to previous study [9]. First of all, the expression values of the lncRNAs and mRNAs were obtained. Then, Spearman correlation coefficient was calculated between the expression values of each of the lncRNA-mRNA pairs across the near native samples JH and the transplanted site samples QG, respectively. The data were preprocessed without special treatment of the lncRNAs or mRNAs expression value. A *P*-value of less than 0.05 was considered statistically significant. The lncRNA-mRNA pairs whose Spearman correlation coefficients was larger than 0.7 in one environment (native environment JH or transplanted site QG), but smaller than 0.5 in the other environment were selected, as these parameters indicated that the lncRNA-mRNA pairs were differentially co-expressed between the two distinct environments [9]. Finally, the network was constructed, in which nodes were lncRNAs or mRNAs. A total of 241 lncRNAs and mRNAs containing 334 relationships were visualized using Cytoscape [71]. The top 20 mRNA nodes which were those with the highest degree were shown. Two sub-networks containing 32 lncRNAs and mRNAs and 56 relationships with most lncRNA-mRNA interactions were presented in detail. Circle nodes represent lncRNAs and square nodes represent mRNAs.

### Construction of water use efficiency (WUE) related differential lncRNA-mRNA co-expression network

By re-analyzing transcriptome expression level associated with water use efficiency (WUE), a WUE related differential lncRNA-mRNA co-expression network (WUE-LMN) was constructed in the current study. First of all, we conducted a matrix correlation between water use efficiency (WUE) and expression level of protein coding mRNAs. Before that, the genes with FPKM of the median value of 0 were dropped out, and 15,495 mRNAs were ultimately used for the further analysis. For each gene, the FPKM value of each individual in QG divided by the FPKM value in JH with all combinations, and then a matrix were obtained. The same process was also performed for water use efficiency (WUE). Following, we performed mantel test between the water use efficiency (WUE) matrix and FPKM matrix using Spearman’s rank correlation method. Furthermore, a Spearman’s rank correlation coefficient was calculated for each gene using a 10,000 permutation test to assess the statistical significance. The detail method was descripted in our previous work [32]. Using this method, 48 genes were identified at the *P* < 0.01 level as candidates genes for WUE alteration in the changing environment under stress [32]. The co-expression of lncRNAs and mRNAs as well as WUE was also visualized using Cytoscape [71]. The Pfam database was used to analyze the potential functions of lncRNAs based on their co-expressed genes.

### Validation of sequence assembly and gene expression from RNA-Seq

In order to validate the sequence quality of this assembly, eight randomly selected lncRNAs were sequenced by PCR sequencing method. RNA sample from one of these individuals was used and reverse-transcribed into the first strand cDNAs for the PCR template. The primers were listed in Additional file 10. All the PCR products were obtained from the same sample. The clear PCR results were blasted against the assembly sequence from RNA-seq using BLASTN. RNA samples from the 18 individuals which passed the quality control were used and reverse-transcribed into the first strand cDNAs for the qPCR quantification. Eight lncRNAs were examined for the relative quantitation of expression level in populations. The qPCR was performed using SYBR Premix Ex Taq (Takara) on ABI7500 (Applied Biosystems). The primers were listed in Additional file 11. According to previous method [32, 40–42], we performed three technical replicates for each template to calculate the average CT value, which were used to evaluate the relative expression level of lncRNAs. Relative expression levels of target lncRNAs among the individuals were determined using the 2 ^–ΔΔCt^ method to calculate the relative quantitation of expression levels with the normalization gene Actin as the endogenous control (The accession number was AT3G53750 from TAIR of the *Arabidopsis* homologue) [42]. The correlation of lncRNA expression estimated by qPCR and RNA-Seq were analyzed using Spearman test with R (Version 3.3.0).

## Additional files

Additional file 1: The sequence of 17610 lncRNAs identified from the population of *M. lutarioriparius. (XLSX 3694 kb)*

Additional file 2: The sequence of 8 lncRNAs obtained from population RNA-seq assembly and the PCR experiment. (XLSX 12 kb)
Additional file 3: The *E*_p_ ratios, *E*_d_ and *E*_d_ ratios for each lncRNA between two environments JH and QG. (XLSX 1967 kb)
Additional file 4: The *E*_p_ ratios, *E*_d_ and *E*_d_ ratios for each mRNA between two environments JH and QG. (XLSX 1784 kb)
Additional file 5: The expression frequency of each lncRNA in population and their different frequency between two environments JH and QG. (XLSX 1136 kb)
Additional file 6: The lncRNA-mRNA pairs in both environments with the correlation coefficient of more than 0.9. (XLSX 234 kb)
Additional file 7: The lncRNA-mRNA pairs with Spearman correlation coefficients larger than 0.7, including 1 052 mRNAs and 1 023 lncRNAs. (XLSX 44 kb)
Additional file 8: The lncRNA-mRNA pairs in the WUE-lncRNA-mRNA co-expression network. (XLSX 75 kb)
Additional file 9: Collection locations and experimental field sites for each of the sampled individuals of the *M. lutarioriparius* species. (XLSX 10 kb)
Additional file 10: List of primer sequence of 8 lncRNAs for the PCR validation experiments. (XLSX 8 kb)
Additional file 11: List of primer sequence of 8 lncRNAs for qPCR validation experiments. (XLSX 9 kb)

## Abbreviations

CT: Cycle threshold
*E*_d_: Expression diversity
*E*_p_: Average expression level
FPKM: Expected number of fragments per kilobase of transcript sequence per millions base pairs sequenced
GO: Gene ontology; indels (deletions and insertions)
JH: Jiangxia in Hubei Province
lncRNA: Long non-coding RNA
QG: Qingyang in Gansu Province
qPCR: Quantitative real-time PCR
WUE: Water use efficiency
